# The Effect of Social Skills Training on Perceived Competence of Female Adolescents with Deafness

**DOI:** 10.5812/ircmj.5426

**Published:** 2013-12-05

**Authors:** Tahereh Soleimanieh Naeini, Farnaz Keshavarzi Arshadi, Nikta Hatamizadeh, Enayatollah Bakhshi

**Affiliations:** 1 Department of Psychology and Education of Exceptional Children, Islamic Azad University- Central Tehran Branch, & Pediatric Neuro-rehabilitation Research Center, University of Social Welfare and Rehabilitation Sciences, Tehran, IR Iran; 2 Department of Clinical Psychology, Islamic Azad University- Central Tehran Branch, Tehran, IR Iran; 3 Pediatric Neuro-rehabilitation Research Center, University of Social Welfare and Rehabilitation Sciences, Tehran, IR Iran; 4 Department of Biostatistics, University of Social Welfare and Rehabilitation Sciences, Tehran, IR Iran

**Keywords:** Adaptation, Psychological, Deafness, Hearing Loss

## Abstract

**Background:**

Although there are considerable researches on effectiveness of social skills training, little information is available on the effects of such training on perceived competence of adolescents with deafness.

**Objectives:**

This study was conducted in special school settings to determine the effects of social skills training on perceived competence of female adolescents with deafness.

**Patients and Methods:**

A prepost quasi-experimental design was used to perform the study. Sixty nine female students with deafness who were enrolled in all of the four different special secondary schools in Tehran, Iran, between 2010 and 2011 participated in this research. Two of four secondary schools were randomly allocated to the intervention group (33 students), and the other two to the control group (36 students). The participants were between 11 and 21 years (Mean = 15.43; SD = 1.89), and more than three fourth of each groups ( i.e. 28 students in each groups) were affected by profound hearing impairment . The intervention group participated in twelve bi-weekly sessions. Pretest and posttest data were collected using the ‘Hearing Impaired Children Self-Image Test’. The questionnaire was filled by an interviewer. This questionnaire asks students about their feeling toward their own competence in domains of cognitive, physical, socio-emotional and communication competence and school adjustment. The data was analyzed by using SPSS software, version 16.

**Results:**

The intervention led to significant improvement in total perceived competence scores of adolescents with deafness (P < 0.001) as well as in three domains of socio-emotional competence (P = 0.003), communication competence (P < 0.001), and school adjustment (P = 0.018).

**Conclusions:**

It is likely that learning social skills in adolescents with deafness would improve their sense of competence, and emotional well being.

## 1. Background

Hearing loss is the most prevalent sensory disability increasing globally (1). Some people are born with hearing loss or lose their hearing ability for different causes (1). Hearing is one of the essential learning tools for acquisition of language skills, interaction with environment, and prerequisite for child’s development (1, 2). Lack of this tool can lead to developmental delay, and lifelong difficulties of individuals with deafness (3). Affected individuals would have difficulties in communication and making social relationships (3). When a person move from childhood to adolescence, many changes in physical, psychological, and social development would occur (4). Adolescence can be a time of increased risk for the onset of a wide range of emotional and behavioral problems, including depression, violent delinquency, and substance abuse (5). Acceptance by peers and the ability to develop and keep close friendships help adolescents improve social skills and social competence, promote self-esteem, and prevent loneliness (6, 7). Those adolescents who have friends tend to be less aggressive, less selfish, more confident, and more involved with school than adolescents with no friends (10). Having no group of friends, may lead to miss out many opportunities of peer interaction, imitation, and learning important lifelong skills (8-10). Friends have been found to be influential in adolescent’s making short-term choices for their lives, such as their appearance and interests (11).

**Figure 1. fig7439:**
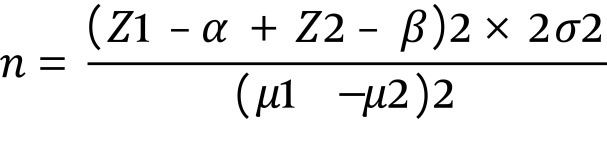
Equation 1 σ2 = 4.25, µ1 = 14.25, µ2 = 15.92

For deaf person who entered adolescence and are not well equipped with necessary tools for this challenging period, it is not surprising to become struggled with feeling of self-consciousness about their deafness and worries around friendships (12).

As a group of students with special needs, children and adolescents with deafness have difficulties in acquiring social skills through daily experiences. These include communication difficulties, low experience in peer interactions (13), as well as low school adaptation, and school achievement (14). Social skills deficit is one of the leading factors which can lead to peer-rejection, isolation, school disengagement, behavior problems, and academic failure in adolescents with deafness (14). Furthermore, this deficit would decrease perceived competence of these students (15). Perceived competence is the way in which a person thinks about his or her ability to cope with all aspects of the environment (16). It helps a person to be intrinsically motivated to learn new skills, feel more in control and even if experiencing failure in different domains, exerts more effort (17); whereas, those who experience lower perceptions of competence, as children and adolescents with deafness, avoid participation, apply little effort, and demonstrate a negative effect in the form of anxiety and low achievement levels (18, 19).

Acquisition of social skills plays an integral role in developing social relationships for children and adolescents and improves overall quality of life (20). It also facilitates acquiring independence in daily life and adapting different situations in life (21).

Emphasis on facilitating promotion of social skills has received increasing attention from psychologists, educators and pediatricians who are dealing with child development in the recent years. Searching PubMed, ERIC and Trip database revealed that while there are many reports on effectiveness of social skills training on adolescents with different types of disabilities, such reports on adolescents with hearing impairment is rather few surprisingly (22, 23), and old and most of them deal with the effects of training on preschool (13, 24-28), and primary school children (15, 18-25).

## 2. Objectives

According to the importance and impact of self competence for mental health and achievements of adolescents with deafness, the aim of this study was to investigate the effects of a social skills training program on perceived competence of female adolescents afflicted with severe and profound deafness.

## 3. Patients and Methods

### 3.1. Participants 

Based on the study of Hatamizadeh et al. (12) and using the following sample size formula, assuming a confidence interval of 95 % and 80 % power, sample size was calculated to be 31 students for each group. There were a total number of sixty nine girls with hearing impairment enrolled in all of the four different special secondary schools in Tehran, Iran, in school year 2010-2011. Two of four secondary schools were randomly allocated to the intervention group (33 students enrolled in this group), and the other two to the control group (36 students enrolled in this group). Considering a sample loss of 10% all of the students of these schools who were studying in 6-8 grades were asked to participate in the research. The inclusion criterion was ‘studying in 6-8 grades at special school settings for female adolescents with deafness’. The exclusion criteria were ‘not willing to participate in research’ and ‘have not signed the informed consent’ on behalf of the students or their parents. The participants were between 11-21 years old (Mean = 15.43; SD = 1.89), and more than three fourth of each group of students (i.e. 28 students) were affected by profound hearing impairment.

### 3.2. Intervention

The intervention group participated in twelve 60-minute bi-weekly sessions, focusing on three major social skills: 1) knowing and respecting self, 2) making friendship, and 3) recognizing one’s emotions and ways to manage negative ones, especially the ‘anger’. The training method consisted of a short explanation by tutor, followed by group discussion and role-playing. The subjects and content of these 12 sessions were as described below.

#### 3.2.1. Session One: An Introduction to Social Skills and the Training Course

Using ‘question and answer technique’ the trainer began the session by asking questions such as; do you know why do you come to school?, what subjects have you studied in school?, what other things do you do at school?, and after receiving answers from the participants, conversation was continued by telling them that. In addition to study subjects you have been mentioned, we want to learn how to live happy, making friends and being kind with each other’. Then the trainer used the same technique to define the term ‘skill’, the phrase ‘social skills’, and the benefits of having social skills, for the participants.

#### 3.2.3. Session two: Recognizing and Respecting Self

In this session the participants were asked to describe themselves by writing their own abilities / inabilities, likes / dislikes and read them in front of the class to see how similar and / or different they are with their classmates. The example of ‘differences and similarities which are existed between pencils of a box of colored pencils’ was used to help them make sense of the notion that people are different from each other, and all off the people have strengths and weaknesses.

#### 3.2.3. Third to fifth sessions: Being Friends and the Skill of Making Friendships.

In these sessions using ‘question and answer technique’ the trainer began the session by these questions: ‘How many friends do you have?, Do you just have friends at school? or you have some friends outside the school as well?, Tell the name of your best friends?. Then the trainer continued the session by asking the students about how would they feel if they did not have any friends?’, ‘How do they feel when they were with their friends?, and do they do for their friends?’ Then the picture stories were introduced to participants. They were asked to tell the related story of each group of pictures, and say if the story showed a friendship or not. For example one of these stories was as follows: Amir and Abbas were neighbors. Amir left the house to buy bread. He saw Abbas fallen on the ground, all his books were scattered on the ground and he was cried out. Amir run to reach him, took his hand and helped him pick up and collect his books.

#### 3.2.4. Sixth to Ninth Sessions: Detecting Facial and Bodily Expressions of Four Basic Feelings of ‘Fear’, ‘Happiness’, ‘Anger’, and ‘Sadness’

The trainer used groups of pictures of people in different situations which made them, sad, angry, happy or fearful, and their emotions were really obvious in the pictures. The participants were asked to tell the story of each group of card and tell what was the person feeling in the picture in that situation, and tell how they knew the emotion of that person by describing the features they had seen in the face, and the whole body of the person in the picture. Then they were asked to tell their own experiences on the same emotions by asking them questions such as ‘Have you ever been sad?, What situations make you sad?, Please give me some examples’.

#### 3.2.5. Tenth to Twelfth Sessions: Different Severities of Anger and Sadness and How to Manage These Negative Emotions, Especially the Anger

Picture stories, asking participants to tell their own experiences on anger and sadness, and their reactions to these situations, and then providing strategies for controlling negative emotions by teacher and exercising them in class were used in these sessions. Also the participants were told that anger could be with different severities, the participants’ physical symptoms of anger expression, and anger range in stories were presented. Then the students were asked what they would do if they were in the place of the main character of the story. Classroom communication mode was total communication. Training materials were chosen from life skills books developed in welfare organization and been used for training the students of regular schools in social skills (29). Topics were chosen according to our participant’s educational needs, and as acquisition of language and social skills is delayed in adolescents with hearing impairment, materials were selected and adopted from books which were ordinary written for students with normal hearing capability who were studying in 4th and 5th grades of regular schools.

### 3.3. Measures

In this prepost quasi-experimental study, the ‘Hearing Impaired Children Self-Image Test’ was used to assess the effects of intervention (28). The questionnaire was filled by an interviewer in a quiet location convenient to participants. This test include 28 items for assessing Iranian hearing impaired students' feeling toward their own competence regarding cognitive, physical, socio-emotional and communication competence and school adjustment, and has been produced on 2008 in Iran. Contents of 23 of 28 items were similar to those in ‘Picture Assessment of Self -Image for Children with Cochlear Implants’, and all of the items were sensitive to the perception and linguistic abilities of Iranian young hearing impairment. The questionnaire included one question requiring a yes/no response, and twenty seven questions elicit ratings of specific social skills using a four-point Likert scale. Of these twenty seven questions, four were elicit cognitive competence, seven representing school adjustment, three showing communication competence, five representing physical competence, and nine pertaining to socio-emotional competence. Scores were ranged between 1- 4 for each domain, and 5- 20 for the total competence score. Face and content validity were measured based on some experts’ opinion. The Cronbach’s alpha coefficient for the instrument was 0.80, and inter-rater reliability of the test was 0.9.

### 3.4. Data Analysis

Statistical analyses were performed using SPSS software, version 16. There was no missing value. Homogeneity of data was assessed using Levene’s test, and normal distribution of data was checked using Kolmogorov Smirnov test. There was no interaction between variables. Data were confirmed to be homogenous and normally distributed. There were no outliers in the research’s data. Confounding factors were controlled in this study by using analysis of covariance, and ANCOVA test was used for examining the significance of changes occurred in interventional group as opposed to any observable change in control group in the same period of time (a 3 month period elapsed between before and after intervention measurements).

### 3.5. Ethical Aspects

This research was approved by the Research Ethics Committee of the University of Social Welfare and Rehabilitation Sciences with the number of 91.801.368 dated 12 Dec 2010. The legal permission for research implementation was gained from Special Education Organization of Tehran, Iran. Informed written consents were obtained from parents and adolescents in this research. Intervention sessions were scheduled in a manner that would not interfere with schools’ training routine schedule. Participants were told to fill free in leaving the study whenever they want to. Nevertheless no participants left the study.

## 4. Results

The participants’ age range was between 11 and 21 years. Most of the participants (53.6 %) aged 15-16 years old, and 81.2 % of them had hearing thresholds more than 90 db on their better ear with hearing aid. There were no statistically significant differences between intervention and comparison groups regarding age groups (P = 0.24) and degrees of hearing loss (P = 0.79). Characteristics of participants in both intervention and control groups are presented in Table 1.

**Table 1.** Characteristics of Participants Among Intervention and Control Groups.

Variable a		Intervention		Control		P-value
		No. (%)	Mean (SD)	No. (%)	Mean (SD)	
**Age, (y)**	11 - 14	10 (30.3)	15.15 (1.734)	7 (19.4)	15.69 (2.012)	0.24
	15 – 17	20 (48.5)		23 (63.9)		
	18 - 21	3 (9.1)		6 (16.7)		
**School grade**	6	6 (18.2)	--	7 (19.4)	--	
	7	16 (48.5)		14 (38.9)		
	8	11 (33.5)		15 (41.7)		
**Hearing loss in better ear, (db)**	55 – 69	1 (3)	96.52 (12.021)	3 (8.3)	97.36 (16.839)	0.79
	70 – 89	4 (12.2)		5 (13.9)		
	90 and up	28 (84.8)		28 (77.8)		

^a^Abbreviations: --, not available

The significance of changes which occurred in perceived competence following intervention was examined by using ANCOVA test.

As presented in Table 2, differences between intervention and control groups for change occurred from before intervention to after intervention measurements in total competence score (P < 0.001) and socio-emotional (P = 0.003), communication competence (P < 0.001) and school adjustment (P = 0.018) domain scores were statistically significant. There were no significant differences between intervention and control groups in other two domains of cognitive and physical competence following the intervention.

**Table 2.** Perceived Competence Scores of Female Adolescents with Deafness in Intervention and Control Groups; Before and After the Intervention.

Domains of Competence	Intervention		Control		P - value^a^
	Mean	SD	Mean	SD
**Cognitive**					
Pre	3.32	0.43	3.13	0.45	NS
Post	3.18	0.42	2.95	0.40	
**Physical**					NS
Pre	2.55	0.46	2.48	0.52	
Post	2.59	0.52	2.43	0.50	
**Socio-emotional**					0.003
Pre	3.13	0.47	2.85	0.42	
Post	3.22	0.41	2.77	0.48	
**School**					0.018
Pre	2.73	0.43	2.89	0.46	
Post	2.93	0.45	2.75	0.38	
**Communication**					< 0.001
Pre	3.20	0.53	2.94	0.62	
Post	3.74	0.25	3.14	0.73	
**Total score**					< 0.001
Pre	14.93	1.69	14.29	1.69	
Post	15.65	1.38	14.07	1.76	

^a^Range of scores was 1- 4 for each domain, and 5- 20 for total scores

## 5. Discussion

The short course social skills training of only 12 sessions led to significant improvement in total perceived competence of deaf adolescents (P < 0.001) as well as in three domains of socio-emotional competence (P = 0.003), communication competence (P < 0.001), and school adjustment (P = 0.018). The acquisition and maintenance of social skills is crucial to the development of social relationship with peers and serves as a basis for the acquisition of a range of critical developmental skills. It is particularly important with the students who are deaf due to their social-emotional difficulties and poor academic performance (27).

Several researches have emphasized on the need for intervention efforts to promote students peer-related social competence, particularly with deaf students who are at risk for continued problems with their social development (27). The results of this study indicated that the training program was effective in promoting student’s social-emotional competence, communication competence, and school adjustment. These findings are in accordance with other studies conducted with different intervention strategies, for addressing social, emotional and communication competences, although the educational settings of schools were not the same as the present study. For example, Monaghan (30) investigated the effects of social skills training on peer interaction among five elementary-age residential children with hearing impairment at Florida school. The results were not consistent for all of the five students i.e. the intervention was effective in increasing sharing behavior for each of the five participants and in decreasing negative interaction for 3 of the 5 participants (30).

In another study, Suarez (31) in Spain reported that the intervention composed of ‘interpersonal problem-solving’ and ‘social skills’ training programs were effective in improving emotional adjustment and social adjustment and self-image as observed by students’ teacher, and also in helping students with profound hearing loss to develop more effective patterns of social behavior in mainstream setting. She asserted that children with deafness became better adjusted when greater attention was given to socio-emotional aspects of the students’ development (30). Also results of Barklage’s (10) investigation on social skills training in Preschoolers with hearing loss in mainstream setting indicated that social interaction of those children was enhanced following the intervention, and the intervention helped them being prepared for the mainstream (10).

Greenberg & Kusché (32) reported that Promoting Alternative Thinking Strategies (PATHS) Curriculum, a school-based preventive intervention for hearing impaired students in self contained classes in regular schools, led to significant improvement in student’s emotional recognition skills, emotional adjustment, social competence, and school performance (32). Because of resource limitations, the current study was performed on girls and in the city of Tehran only. This could impose limitations on generalizing the results to boys and other areas, necessitating further studies in male adolescents and in bigger scales.

This research confirmed the effectiveness of social skills training courses in increasing competence of adolescents with deafness in special settings. However, further research on designing more effective training programs is warranted to confirm the importance of our results.
